# Size-Dependent Photodynamic Anticancer Activity of Biocompatible Multifunctional Magnetic Submicron Particles in Prostate Cancer Cells

**DOI:** 10.3390/molecules21091187

**Published:** 2016-09-06

**Authors:** Kyong-Hoon Choi, Ki Chang Nam, Leszek Malkinski, Eun Ha Choi, Jin-Seung Jung, Bong Joo Park

**Affiliations:** 1 Plasma Bioscience Research Center, Kwangwoon University, 20 Kwangwoongil, Nowon-gu, Seoul 01897, Korea; solidchem@kw.ac.kr (K.H.C.); ehchoi@kw.ac.kr (E.H.C.); 2 Department of Medical Engineering, Dongguk University College of Medicine, Gyeonggi-do 10326, Korea; kichang.nam@gmail.com; 3 Advanced Materials Research Institute, University of New Orleans, New Orleans, LA 70148, USA; lmalkins@uno.edu; 4 Department of Electrical & Biological Physics, Kwangwoon University, 20 Kwangwoongil, Nowon-gu, Seoul 01897, Korea; 5 Department of Chemistry, Gangneung-Wonju National University, Gangneung 25457, Korea

**Keywords:** biocompatible multifunctional submicron particles, hematoporphyrin, folic acid, cytotoxicity, photodynamic anticancer therapy, magnetic resonance imaging

## Abstract

In this study, newly designed biocompatible multifunctional magnetic submicron particles (CoFe_2_O_4_-HPs-FAs) of well-defined sizes (60, 133, 245, and 335 nm) were fabricated for application as a photosensitizer delivery agent for photodynamic therapy in cancer cells. To provide selective targeting of cancer cells and destruction of cancer cell functionality, basic cobalt ferrite (CoFe_2_O_4_) particles were covalently bonded with a photosensitizer (PS), which comprises hematoporphyrin (HP), and folic acid (FA) molecules. The magnetic properties of the CoFe_2_O_4_ particles were finely adjusted by controlling the size of the primary CoFe_2_O_4_ nanograins, and secondary superstructured composite particles were formed by aggregation of the nanograins. The prepared CoFe_2_O_4_-HP-FA exhibited high water solubility, good MR-imaging capacity, and biocompatibility without any in vitro cytotoxicity. In particular, our CoFe_2_O_4_-HP-FA exhibited remarkable photodynamic anticancer efficiency via induction of apoptotic death in PC-3 prostate cancer cells in a particle size- and concentration-dependent manner. This size-dependent effect was determined by the specific surface area of the particles because the number of HP molecules increased with decreasing size and increasing surface area. These results indicate that our CoFe_2_O_4_-HP-FA may be applicable for photodynamic therapy (PDT) as a PS delivery material and a therapeutic agent for MR-imaging based PDT owing to their high saturation value for magnetization and superparamagnetism.

## 1. Introduction

During the past few decades, spinel ferrite (MFe_2_O_4_) materials have been widely investigated because of their excellent magnetic and electrical properties [1] and their potential applications in different technological fields [2] and in biomedicine [3]. Among the various spinel ferrite materials, CoFe_2_O_4_ has received particular attention owing to its excellent chemical stability, good mechanical hardness, high coercivity, moderate saturation-magnetization, positive anisotropy constant, large magnetostrictive coefficient, wear resistance, and electrical insulation [4,5,6,7]. These properties, along with their great physical and chemical stability, make CoFe_2_O_4_ nanoparticles suitable for magnetic recording and biomedical applications. Importantly, CoFe_2_O_4_ nanoparticles are among the most promising candidates for biomedical applications, including magnetic resonance imaging (MRI), magnetic fluid hyperthermia, magnetic separation, biosensors, and targeted and controlled drug delivery [8,9,10,11,12,13,14].

Although CoFe_2_O_4_ nanoparticles have various practical applications, recent studies in biomedical fields have focused on the fabrication of multifunctional nanoparticles with various enhanced properties. To improve the properties of these nanoparticles, many research groups have reported various synthetic methods, such as modification of the surface with specific functional groups that enable conjugation and specific uptake of circulating nanoparticles by organs and subsequent delivery of the drugs into the targeted lesions [15,16,17,18]. Modifications have also been applied to provide strong luminescence or MRI signal in a visible range to enable clinicians to track the nanocarrier system [19,20,21]. Among these various biofunctional groups, hematoporphyrin (HP) is one of the most popular photosensitizers (PSs) and photodynamic therapy (PDT) agents due to its unique properties, including high photo-activity, strong photodynamic efficiency, low toxicity, and fast clearance [22]. These novel properties allow HP to be widely used in medical, biological, and environmental applications. Therefore, the emerging technology of bonding of PDT agents to magnetic materials has become important because these multifunctional materials may have applications in the treatment of various types of cancer cells in vitro and in vivo. In this regard, we previously reported the development of multifunctional magnetic submicron particles conjugated with PSs and vancomycin as photodynamic inactivation agents for targeting of bacteria and capturing or removing pathogens from sites of contamination [23]. However, studies performed to date have not sufficiently investigated photodynamic cancer therapy using magnetic particles. In addition, it is important to confirm the anticancer activity of multifunctional particles of different particle sizes to determine the optimal particle size and dose.

Therefore, in this study, we focused on comparing the photodynamic anticancer efficiencies of various sizes of CoFe_2_O_4_ particles conjugated with HP and folic acid (FA) molecules (CoFe_2_O_4_-HP-FA). To this end, we fabricated CoFe_2_O_4_-HP-FA with well-defined sizes by a simple surface modification process that functionalized the CoFe_2_O_4_ particles by coating them with HP and conjugation with FA as previously described [24,25]. We then examined the capacity of the CoFe_2_O_4_-HP-FA for specific targeting and killing of cancer cells and evaluated their particle size-dependent cytotoxicity and photodynamic anticancer activity. In addition, we performed in vitro MR-imaging with and without cells and analyzed apoptotic death in prostate cancer cells to confirm the potential clinical applications of CoFe_2_O_4_-HP-FA.

## 2. Results and Discussion

### 2.1. Characterization of CoFe_2_O_4_-HP-FA

**Figure 1:**
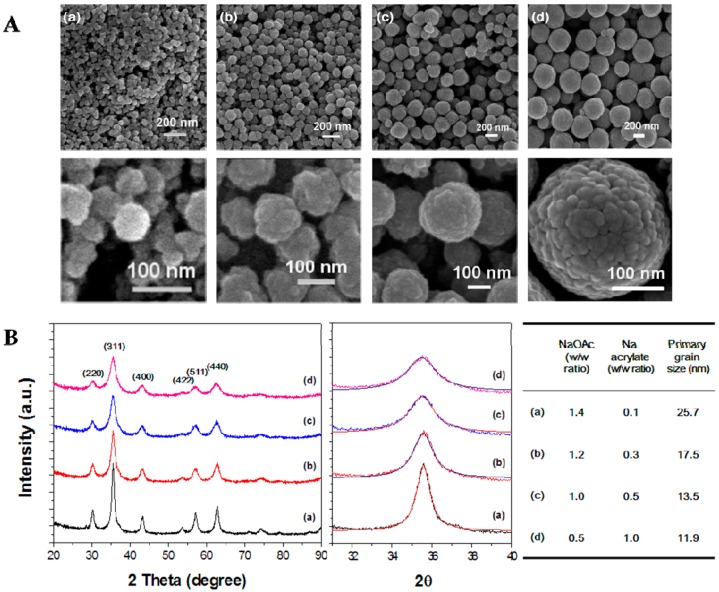
Morphology and crystal structure of CoFe_2_O_4_ submicron particles with controlled external size: (**A**) FE-SEM images of CoFe_2_O_4_ particles with controlled external size synthesized using different volume ratios of V_EG_/V_DEG_: (a) 5/15, (b) 10/10, (c) 15/5, and (d) 20/0. Other experimental parameters were kept constant (NaOAc = 1.0 g, Na acrylate = 0.5 g, temperature = 200 °C, and time = 10 h); (**B**) XRD patterns of CoFe_2_O_4_ particles with tunable grain sizes obtained using different mass ratios of NaOAc/Na acrylate (*w*/*w*): (a) 1.4/0.1, (b) 1.2/0.3, (c) 1.0/0.5, and (d) 0.5/1.0. Other experimental parameters were kept constant (EG = 15 mL, DEG = 5 mL, temperature = 200 °C, and time = 10 h).

The morphologies and crystal structures of CoFe_2_O_4_ submicron particles were investigated using field emission scanning electron microscopy (FE-SEM; SU-70, Hitachi, Tokyo, Japan) and X-ray diffractometry (XRD; X′ Pert Pro MPD, PANalytical, Almelo, The Netherlands). FE-SEM images of CoFe_2_O_4_ particles with controlled external sizes are shown in Figure 1A. All particles exhibited spherical morphology with rough surfaces and were remarkably uniform with a narrow size distribution. The external size of the CoFe_2_O_4_ particles was precisely controlled from 60 to 335 nm by simply increasing the volume ratio of V_EG_/V_DEG_. The mean diameter of the prepared CoFe_2_O_4_ particles was directly proportional to the volume ratio of V_EG_/V_DEG_ during the solvothermal reaction. When the ratio of V_EG_/V_DEG_ was varied from 5/15 to 10/10, 15/5, and 20/0, the diameter of the resulting superstructured CoFe_2_O_4_ particles was 60, 133, 245, and 335 nm, respectively. Moreover, high-magnification FE-SEM images clearly showed that the individual spheres were composed of irregular nanograins with a size of approximately 15 nm (bottom images of Figure 1A).

To obtain monodispersed CoFe_2_O_4_ particles with tunable grain sizes, we designed a modified synthetic route by controlling the NaOAc/Na acrylate ratio (*w*/*w* in grams). The size variation of CoFe_2_O_4_ particle grains was confirmed by XRD pattern analysis (Figure 1B). The positions and relative intensities of all diffraction peaks were well matched with the cubic spinel structure of CoFe_2_O_4_ particles, which is in good agreement with the values reported in the literature (JCPDS Card No. 22-1086). The diffraction peaks from CoFe_2_O_4_ occurred at Bragg angles of 30.2°, 35.6°, 43.3°, 53.6°, 57.1°, and 62.7°, representing reflections from the (220), (311), (400), (422), (511), and (440) lattice planes, respectively. To estimate the grain size according to the mass ratio of NaOAc/Na acrylate, the observed diffraction peak profiles (2θ = 35.6°) were reasonably well fitted by a convolution of Lorentzian functions (middle graph of Figure 1B). The average grain size was calculated based on Scherrer’s equation. With the increase in the mass ratio of Na acrylate, the average size of the primary crystallites decreased from 25.7 to 17.5, 13.5, and 11.9 nm, respectively. Therefore, the grain size of CoFe_2_O_4_ particles decreased gradually with increasing volume ratio of Na acrylate. Additionally, the crystalline characteristics of CoFe_2_O_4_ particles increased as the primary grain size increased. These two experimental results suggest that both the external size and grain size of CoFe_2_O_4_ particles could be adjusted easily by controlling the *V*_EG_/*V*_DEG_ and NaOAc/Na acrylate ratios.

**Figure 2:**
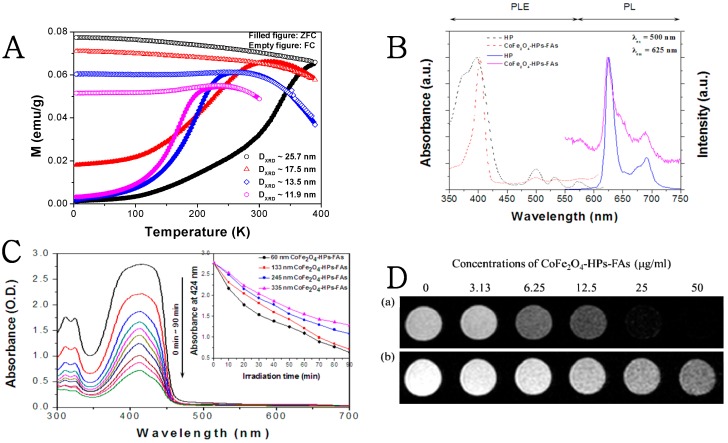
Magnetic and optical properties of multifunctional magnetic particles: (**A**) Temperature dependence of magnetic susceptibility of monodispersed CoFe_2_O_4_ particles in zero-field cooled (ZFC) and field-cooled (FC) conditions measured using a SQUID magnetometer at an applied field of 5 kOe; (**B**) PL and PLE spectra of pure HP and CoFe_2_O_4_-HP-FA in THF. The excitation and detection wavelengths were 500 and 625 nm for PL and PLE spectra, respectively; (**C**) Irradiation time-dependent UV-Vis spectra of DPBF in THF solution with CoFe_2_O_4_-HP-FA excited by the Xe lamp. The inset represents the absorption OD of DPBF in THF at 435 nm as a function of irradiation time. In the inset, (a) DPBF only with the light, (b) DPBF with CoFe_2_O_4_-HP-FA without the light, and (c) DPBF with CoFe_2_O_4_-HP-FA with the light are shown; (**D**) MR (T_2_-weighted) images of CoFe_2_O_4_-HP-FA and PC-3 cells treated with CoFe_2_O_4_-HP-FA. (a) Different concentrations of 60-nm CoFe_2_O_4_-HP-FA. (b) PC-3 cells treated with 60-nm CoFe_2_O_4_-HP-FA. Cells treated with different concentrations of 60-nm CoFe_2_O_4_-HP-FA were incubated for 2 h in the dark before acquisition of MR images.

The magnetic properties of monodispersed CoFe_2_O_4_ particles with tunable grain size were investigated using a superconducting quantum interference device (SQUID; MPMS-5S, Quantum Design, San Diego, CA, USA), as shown in Figure 2A. The temperature characteristics of the magnetization at a constant field of 5 kOe were typical of superparamagnetic materials. The field-cooled (FC) magnetization curves were nearly constant, whereas the curves for the samples cooled in the demagnetized state showed marked maxima, corresponding to the blocking temperature above which superparamagnetism occurred. The blocking temperatures for the smallest nanograins sized 11.9 and 13.5 nm were 245 and 275 K, respectively, which precludes them from being used in medical applications. However, the blocking temperatures for the particles built from larger grains (17.5 and 25.7 nm) were close to RT and above. These results also provide evidence for good separation of nanograins inside the super-structured particles (clusters), as direct contact between aggregating grains would increase the magnetic moment of the effectively larger grains and thus prevent thermal excitation from randomizing magnetic moment directions. Notably, superparamagnetism of magnetic particles is critical for medical applications because this feature prevents aggregation of large magnetic clusters, which occurs for particles with permanent magnetic moments.

Using a wet chemical process, size-controlled CoFe_2_O_4_ particles were easily bonded with HP molecules after plasma treatment. As shown in Figure 2B, the photoluminescence (PL) and photoluminescence excitation (PLE) spectra of CoFe_2_O_4_-HP-FA exhibited the same characteristics as pure HP molecules in tetrahydrofuran (THF). The peak at 400 nm in both PLE spectra was attributed to the Soret band of HP, and the peaks at 500, 532, and 568 nm was attributed to the Q bands of HP. At the excitation wavelength of 500 nm, pure HP produced two strong emission peaks at 623 and 690 nm, and CoFe_2_O_4_-HP-FA yielded slightly blue-shifted peaks at 619 and 685 nm. The blue-shifted emission peaks were attributed to strong bonding between HP and the magnetic CoFe_2_O_4_ particles. To understand the bonding property of the carboxyl groups of HP and FA and the metal ions of the CoFe_2_O_4_ particle, the FT-IR spectra of all samples were compared as shown in Figure S1. Based on this result, we concluded that the carboxyl terminal groups of HP formed cation-carboxylate complexes as a result of chemical coordination bonding between the cation and the carboxylate; some molecules would also be expected to exist in the protonated carboxyl state. The IR spectrum of FA bonded to CoFe_2_O_4_ particles showed similar results after the interface bonding reaction, demonstrating that the protonated carboxyl group disappeared and that new bands appeared at 1570 and 1430 cm^−1^ (Figure S1B).

The concentration of HP molecules bonded to the surfaces of the CoFe_2_O_4_ particles depended on the weight and size of the CoFe_2_O_4_ particles as a substrate. As the size of the particles decreased, the number of HP molecules bonded to the surfaces of the CoFe_2_O_4_ particles increased under the same weight conditions due to the increase in surface area of the whole particles. When the density of CoFe_2_O_4_ particles was fixed (ρ = 5.3 g/cm^3^), approximately 50 μg of CoFe_2_O_4_ particles with various sizes (60, 133, 245, and 335 nm) would contain 7.4 × 10^10^, 6.9 × 10^9^, 1.1 × 10^9^, and 4.5 × 10^8^ primary particles with surface areas of 8.4, 3.8, 2.1, and 1.6 cm^2^, respectively. Practically, the number of calculated HP molecules was inversely proportional to the size of the CoFe_2_O_4_ particles determined by analysis of the UV-VIS absorption spectra. When the particle size varied (60, 133, 245, and 335 nm), the weight of the HP molecules bonded to the surfaces of the CoFe_2_O_4_ particles was 5.52, 5.24, 4.84, and 3.17 μg, respectively, under the same weight conditions (50 μg of CoFe_2_O_4_-HP particles). Similarly, the weight of the FA molecules bonded to the surfaces of the CoFe_2_O_4_ particles was 2.64, 2.23, 2.07, and 1.68 μg according to particle sizes (60, 133, 245, and 335 nm), respectively, under the same weight conditions (50 μg of particles). Therefore, the smallest particle complex was expected to exhibit the highest generation efficiency for singlet oxygen due to the increase in dose.

Singlet oxygen generation from CoFe_2_O_4_-HP-FA was confirmed by indirect detection of DPBF photodegradation. As a specific singlet oxygen quencher, DPBF readily undergoes a 1,4-cycloaddition reaction with singlet oxygen to form endoperoxides. DPBF then further irreversibly decomposes into 1,2-dibenzoylbenzene [26]. The reaction can easily be followed by measuring the decrease in the optical density of DPBF absorption at 424 nm [27]. Figure 2C illustrates the changes in the absorption spectra of DPBF, which depended on the reaction time with irradiation and the size of the CoFe_2_O_4_-HPs-FAs particles. Notably, under excitation with the Xe lamp, the absorption intensity decreased as the irradiation time increased according to the UV-VIS spectra of DPBF in the THF solution with CoFe_2_O_4_-HPs-FAs. Moreover, the photodegradation efficiency of DPBF increased as the particle size of CoFe_2_O_4_-HPs-FAs decreased to 335, 245, 133, and 60 nm. These results were consistent with the change in the number of conjugated HP molecules as the particle size decreased.

### 2.2. In Vitro Magnetic Resonance (MR) Imaging Measurement

To evaluate the potential applications of CoFe_2_O_4_-HP-FA as a contrast agent for MR-imaging, in vitro MR-imaging contrasts were detected in CoFe_2_O_4_-HPs-FAs samples and PC-3 cells treated with various concentrations (0, 3.13, 6.25, 12.5, 25, and 50 μg/mL) of 60 nm CoFe_2_O_4_-HP-FA using a 3.0 T human clinical scanner at RT as previously described [24,25]. Figure 2D shows that all CoFe_2_O_4_-HPs-FAs efficiently shortened the transverse relaxation time (T_2_) and significantly decreased signal intensity in T_2_-weighted MR images of PC-3 cells, even at low concentrations. Additionally, the T_2_ phantom changed significantly in signal intensity with increasing CoFe_2_O_4_-HP-FA concentration in PC-3 cells. The strong T_2_ shortening effect, which likely resulted from stronger magnetic susceptibility caused by agglomeration, led to spin dephasing and a substantial decrease in the MR-imaging signal, thereby generating a “darkening” contrast in T_2_-weighted MR images.

### 2.3. Biocompatibility of the Multifunctional Magnetic Submicron Particles

The biocompatibility of biomaterials is critical for clinical applications. Therefore, we evaluated the cytotoxicity of different sizes and concentrations of CoFe_2_O_4_-HP-FA in fibroblasts (L-929 cells) and prostate cancer cells (PC-3 cells) to confirm their biocompatibility as recommended in International Standard ISO10993-5 [24,25,28]; we repeated this analysis because it is essential for clinical application.

**Figure 3:**
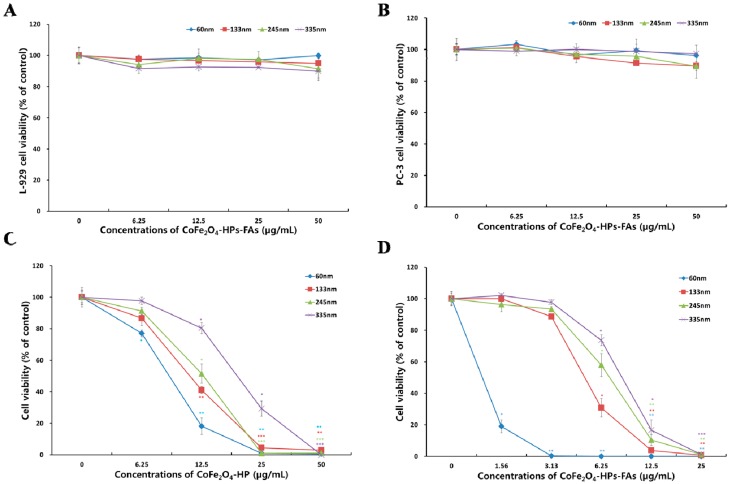
Cytotoxicity and photodynamic anticancer activity of multifunctional magnetic particles in PC-3 cells. (**A**) Cytotoxicity in fibroblast (L-929 cells) and (**B**) prostate cancer (PC-3 cell). Cells were cultured with different concentrations of CoFe_2_O_4_-HP-FA for 24 h at 37 °C in the dark; (**C**) Photodynamic anticancer activity of each size of CoFe_2_O_4_-HP in PC-3 cells; (**D**) Photodynamic anticancer activity of each size of CoFe_2_O_4_-HP-FA in PC-3 cells. Cells were incubated with different concentrations of CoFe_2_O_4_-HP and CoFe_2_O_4_-HP-FA for 2 h in the dark prior to irradiation for 30 min. Data are expressed as the mean ± standard deviation (*n* = 6) and were analyzed by Student’s *t*-test. Statistical significance was defined as *p* < 0.05 (* *p* < 0.05, ** *p* < 0.005, *** *p* < 0.001 vs. control at the same time).

Figure 3A,B show the viability of L-929 and PC-3 cells after treatment with CoFe_2_O_4_-HP-FA of different sizes for 24 h. All sizes of CoFe_2_O_4_-HP-FA did not exhibit cytotoxicity in L-929 and PC-3 cells under our experimental conditions at concentrations of 0 to 50 μg/mL. As shown in Figure 3A,B, the viability of both cell types was higher than 95%. The cytotoxicity tests demonstrated that CoFe_2_O_4_-HP-FAs were not cytotoxic and exhibited good biocompatibility in fibroblasts (L-929) and prostate cancer cells (PC-3 cells), regardless of their size; therefore, all of our CoFe_2_O_4_-HP-FAs could be safely applied for in vivo experiments and clinical applications in photodynamic cancer therapy.

### 2.4. In Vitro Photodynamic Anticancer Activities

To compare the photodynamic anticancer activity of different sizes of CoFe_2_O_4_-HP and CoFe_2_O_4_-HP-FA in prostate cancer cells, we used PC-3 cells, which are commonly used for studies of metastatic prostate cancer and are known to express prostate-specific membrane antigen (PSMA) as previously described [24,25,27,28,29,30].

Figure 3C,D show the photodynamic anticancer activity of these particles according to the viability of PC-3 cells normalized to that of the control. PC-3 cell viability was lower than 3% in the presence of 50 μg/mL CoFe_2_O_4_-HP, regardless of particle size, and lower than 5% in the presence of 25 μg/mL CoFe_2_O_4_-HP for all particle sizes except 335 nm (29.4% ± 4.8%). At a concentration of 12.5 μg/mL CoFe_2_O_4_-HP, the viability was 18.2% ± 5.2% for particles measuring 60 nm (*p* < 0.003), 41.1% ± 2.2% for particles measuring 133 nm (*p* < 0.004), 51.5% ± 6.2% for particles measuring 245 nm (*p* < 0.02), and 80.4% ± 3.5% for particles measuring 335 nm (*p* < 0.01). These results demonstrate that CoFe_2_O_4_-HP had significantly different anticancer activity depending on the concentration in inverse proportion to particle size, despite the lack of targeting molecules for cancer cells.

Additionally, the viability of PC-3 cells in the presence of 25 μg/mL CoFe_2_O_4_-HP-FA was lower than 1.2%, regardless of particle size. In particular, the viability of cells in the presence of 12.5 μg/mL CoFe_2_O_4_-HP-FA was 0% ± 0% for particles measuring 60 nm (*p* < 0.002), 3.7% ± 0.9% for particles measuring 133 nm (*p* < 0.001), 10.4% ± 3.5% for particles measuring 245 nm (*p* < 0.004), and 16.6% ± 6.6% for particles measuring 335 nm (*p* < 0.007). Moreover, the viability of cells in the presence of 6.25 μg/mL CoFe_2_O_4_-HP-FA was 0% ± 0.4% for particles measuring 60 nm (*p* < 0.002), 30.8% ± 5.9% for particles measuring 133 nm (*p* < 0.01), 58.0% ± 7.3% for particles measuring 245 nm (*p* < 0.04), and 73.6% ± 3.4% for particles measuring 335 nm (*p* < 0.02). For particles with a size of 60 nm, the cell viability in the presence of 3.13 or 1.56 μg/mL CoFe_2_O_4_-HP-FA was 0.3% ± 0.1% (*p* < 0.002) and 19.0% ± 0.1% (*p* < 0.006), respectively. These results demonstrate that CoFe_2_O_4_-HP and CoFe_2_O_4_-HP-FA had significantly different anticancer effects in inverse proportion to particle size. As the concentration of CoFe_2_O_4_-HP and CoFe_2_O_4_-HP-FA increased, both overall and as the size decreased, the viability of PC-3 cells decreased significantly. These results showed that smaller CoFe_2_O_4_-HP and CoFe_2_O_4_-HP-FA particles exhibited stronger phototoxic effects than larger particles in PC-3 cells. Furthermore, these results were also consistent with the observed effects of different concentrations of HP in CoFe_2_O_4_-HP and CoFe_2_O_4_-HP-FA having different sizes, as described above. High concentrations of HP on the surface of CoFe_2_O_4_-HP and CoFe_2_O_4_-HP-FA could easily generate a large amount of singlet oxygen, which acts as a key molecule in photodynamic anticancer activity by inducing apoptotic cell death during LED irradiation [29,30].

In contrast, comparison of the photodynamic anticancer activity of CoFe_2_O_4_-HP and CoFe2O4-HP-FA indicated that the anticancer activity of 6.25 μg/mL CoFe_2_O_4_-HP-FA was 4.4–11.5-fold higher than that of CoFe_2_O_4_-HP and that CoFe_2_O_4_-HP-FA exhibited more potent phototoxic effects than CoFe_2_O_4_-HP in PC-3 cells when used at relatively low concentrations (1.56 and 3.13 μg/mL). These results suggest that enhanced cellular uptake of CoFe_2_O_4_-HP-FA into PC-3 cells occurred through PSMA expressed by these cells, leading to FA receptor-mediated endocytosis based on the increased phototoxic activity compared with that of CoFe_2_O_4_-HP [31].

### 2.5. Cellular Uptake and Intracellular Localization

**Figure 4:**
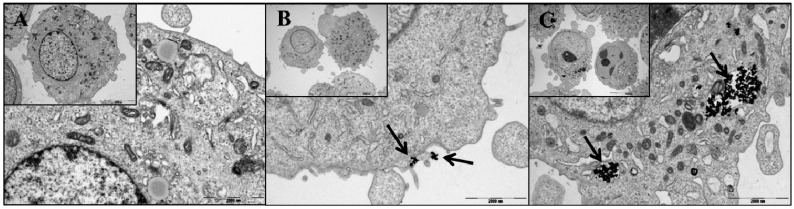
TEM images of PC-3 cells. (**A**) Cells only; (**B**) Cells with 60-nm CoFe_2_O_4_-HP; (**C**) Cells with 60-nm CoFe_2_O_4_-HP-FA. Cells were incubated with 6.25 μg/mL CoFe_2_O_4_-HP or CoFe_2_O_4_-HP-FA for 2 h in the dark. Scale bar: 2 μm.

To compare the cellular uptake and intracellular distribution of our particles without (CoFe_2_O_4_-HP) and with the FA targeting molecule (CoFe_2_O_4_-HP-FA) in prostate cancer cells (PC-3 cells), we used TEM to visualize the cellular uptake and intracellular distribution of the particles, as shown in Figure 4. The images clearly showed differences in cellular uptake and intracellular localization between CoFe_2_O_4_-HP and CoFe_2_O_4_-HP-FA. As shown in Figure 4C, CoFe_2_O_4_-HP-FA particles were located mostly in the cytoplasm of PC-3 cells and had very strong photodynamic anticancer effects. However, CoFe_2_O_4_-HP particles without FA were located only on the surface of the cell membrane (Figure 4B) and therefore exhibited lower photodynamic anticancer activity than CoFe_2_O_4_-HP-FA. These TEM images suggest that FA could enhance the cellular uptake and intracellular localization of CoFe_2_O_4_-HP-FA via binding to PSMA because PC-3 cells, which are known to be negative for the FA receptor (FR), express PSMA, thus leading to FR-mediated endocytosis [31]. This may explain the observed phototoxic effects of CoFe_2_O_4_-HP-FA in PC-3 cells, consistent with the photodynamic anticancer activity shown in Figure 3C,D.

### 2.6. Apoptotic DNA Fragmentation and Caspase-3 and -7 Enzyme Activity Assays

**Figure 5:**
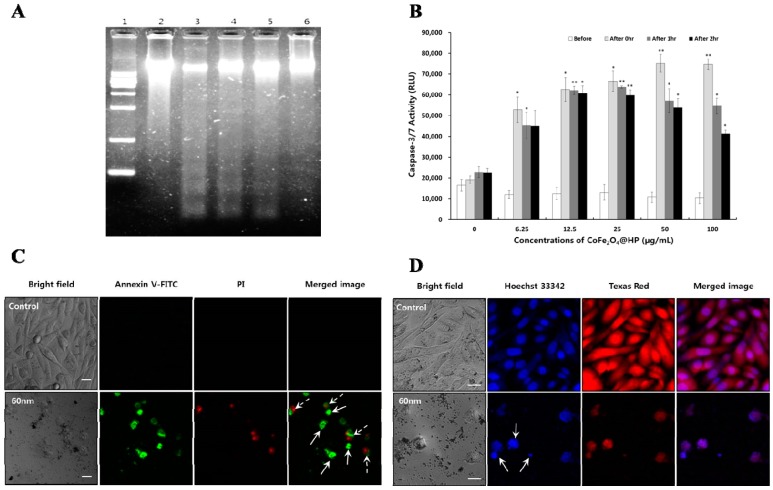
Apoptotic DNA fragmentation, Capase-3/7 activity, cell membrane translocation, and nuclear fragmentation in PC-3 cells. (**A**) Apoptotic DNA fragmentation in PC-3 cells. Apoptosis was induced by irradiation for 30 min after treatment with each size of CoFe_2_O_4_-HP-FA at 6.25 µg/mL in PC-3 cells. Lane 1; DNA size marker (1 kb), Lane 2; control, Lane 3; 60-nm MNPs, Lane 4; 133-nm MNPs, Lane 5; 245-nm MNPs, Lane 5; 335-nm MNPs; (**B**) Caspase-3/7 activity was evaluated after treatment with 60-nm CoFe_2_O_4_-HP-FA for 2 h without irradiation and at 0, 1, and 2 h post irradiation. Data are expressed as the mean ± standard deviation (*n* = 6) and were analyzed by Student’s *t*-test. Statistical significance was defined as *p* < 0.05 (* *p* < 0.05, ** *p* < 0.005 vs. control at the same time); (**C**) Confocal fluorescence images of Annexin V-FITC (green) staining indicating cell membrane inversion and PI (red) for nuclear staining in PC-3 cells at 12 h post irradiation. Apoptosis was induced by irradiation for 30 min after treatment with 60-nm CoFe_2_O_4_-HP-FA at 3.13 µg/mL for 2 h. Control cells were incubated without MNPs. Arrows show apoptotic cells (line arrows; early stage of apoptosis, dashed arrows; late stage of apoptosis); (**D**) Confocal fluorescence images for nuclear fragmentation (line arrows); Hoechst 33342 (blue) shows nuclei and Texas Red (red) shows whole cells in PC-3 cells at 12 h post irradiation. Apoptosis was induced by irradiation for 30 min after treatment with 60-nm CoFe_2_O_4_-HP-FA at 3.13 µg/mL for 2 h. Control cells were incubated without MNPs. Scale bar represents 25 µm.

Internucleosomal DNA fragmentation is a hallmark of apoptosis in cells, resulting from activation of the caspase-3/7 cascade [32]. Therefore, we examined apoptotic cell death resulting from DNA fragmentation after irradiation. As shown in Figure 5A, no DNA ladder formation was observed in the untreated control; however, a typical DNA ladder pattern was progressively observed in PC-3 cells treated with 6.25 μg/mL CoFe_2_O_4_-HPs-FAs (measuring 60, 133, 245, and 335 nm). DNA fragmentation was also inversely proportional to the size of the CoFe_2_O_4_-HP-FA, and this result was consistent with the observed anticancer activity of 6.25 μg/mL CoFe_2_O_4_-HP-FA. Thus, the increased anticancer activity observed in Figure 3D resulted from induction of apoptotic DNA fragmentation, as shown in Figure 5A.

Caspase enzymes are cytoplasmic aspartate-specific cysteine proteases that play a central role in apoptosis in response to pro-apoptotic signals. In particular, caspase-3 and -7, known as the effector caspases, act further downstream and direct cellular breakdown through cleavage of structural proteins. Activation of caspase-3 and -7 is a hallmark of apoptosis [30,32]. Therefore, we evaluated the effects of 60-nm CoFe_2_O_4_-HP-FA on photo-induced apoptosis after irradiation using caspase-3 and -7 enzyme activity assays.

Figure 3C,D show concentration-dependent increases in photodynamic anticancer activity in PC-3 cells after irradiation, mainly as a result of significantly increased caspase-3 and -7 enzyme activity. Figure 5B shows the caspase-3 and -7 activity before and after irradiation followed by treatment with various concentrations of 60-nm CoFe_2_O_4_-HP-FA particles. These results indicate that irradiation after treatment with CoFe_2_O_4_-HP-FA effectively activated caspase-3 and -7 in PC-3 cells, leading to apoptotic cell death.

### 2.7. Morphological Detection of Apoptotic Cell Death

Next, we morphologically detected apoptotic cells using Annexin V-FITC and PI staining to evaluate the predominant morphological changes through which CoFe_2_O_4_-HP-FA caused apoptotic cell death; this assay can easily evaluate apoptotic cell death using Annexin V-FITC, which binds to the translocated membrane phosphatidylserine of apoptotic cells, and PI, which reveals the nuclear deformation of apoptotic cells. Figure 5C shows early- and late-stage apoptosis by double-staining with Annexin V-FITC and PI after irradiation for 30 min. Importantly, cells stained by Annexin V-FITC and PI were not detected in the untreated control, demonstrating that cell death induced by light irradiation after treatment with CoFe_2_O_4_-HP-FA may occur through apoptosis for all sizes of CoFe_2_O_4_-HP-FA.

We also observed nuclear fragmentation in PC-3 cells using Hoechst 33342 dye, as shown in Figure 5D. CoFe_2_O_4_-HP-FA-treated PC-3 cells showed more condensed and granular apoptotic nuclear bodies as compared with the control, demonstrating that light irradiation after treatment with CoFe_2_O_4_-HP-FA induced apoptosis in prostate cancer cells (PC-3 cells).

Taken together, findings were consistent with the observed anticancer activity (Figure 3D). These effects and the resulting cell death may be mediated via apoptosis induced by irradiation after CoFe_2_O_4_-HP-FA treatment.

## 3. Materials and Methods

### 3.1. Preparation of CoFe_2_O_4_-HP-FA Submicron Particles with Various Particle Sizes

CoFe_2_O_4_ submicron particles were prepared using a method similar to that reported previously [33]. In a typical procedure for synthesis of CoFe_2_O_4_ particles, FeCl_3_·6H_2_O (4/3 mmol), CoCl_2_·2H_2_O (2/3 mmol), NaOAc, and Na acrylate were dissolved in a mixture of ethylene glycol (EG) and diethylene glycol (DEG) in a beaker under magnetic stirring. After 30 min, the as-formed viscous slurry was transferred into a Teflon-lined stainless-steel autoclave with a capacity of 80 mL. The autoclave was heated to and maintained at 200 °C for 10 h and then allowed to cool to room temperature (RT). The ratio of NaOAc/Na acrylate (*w*/*w* in grams) controlled the grain size of the CoFe_2_O_4_ particles. For example, ratios of 1.4/0.1, 1.2/0.3, 1.0/0.5, and 0.5/1.0 led to synthesis of CoFe_2_O_4_ particles with average grain sizes of 25.7, 17.5, 13.5, and 11.9 nm, respectively. Similarly, the volume ratio of EG to DEG (*V*_EG_/*V*_DEG_) controlled the external particle size of the CoFe_2_O_4_ particles.

To introduce a large amount of PS molecules, the surfaces of CoFe_2_O_4_ particles having different sizes were treated with microdielectric barrier discharge (DBD) plasma for 30 min under an electrical discharge power of less than 3 W (0.7 kV, 5 mA, and phase angle of ~1 radian). After plasma treatment, the optical functionality on magnetic particles was provided by a wet chemical process with HP, similar to the method described in our previous report [27]. The concentration of HP and FA molecules bonded to the surfaces of CoFe_2_O_4_ nanoparicles was estimated by using UV-visible absorption spectroscopy. The comparative absorption ODs at 500 nm (HP: 2.02 × 10^−4^ M) and 283 nm (FA: 1.1 × 10^−4^ M) of the reference solution and the remaining solution obtained after removing the magnetic aggregation in the magnetic particle solution result the reacted concentration of HP and FA molecules with the particles.

### 3.2. Detection of Singlet Oxygen

Degradation of 1,3-diphenylisobenzofuran (DPBF) as a singlet oxygen quencher was studied in the presence of CoFe_2_O_4_-HP-FA. In the photochemical experiment, 3.0075 mL of THF solution containing CoFe_2_O_4_-HP-FA (1 mg) and DPBF (4.61 × 10^−8^ M) was introduced into a 1-cm quartz cell under dark conditions. The experiments were performed by irradiating the samples with a Xe lamp (150 W, Abet Technologies, Milford, CT, USA). A 480-nm glass cut-off filter was used to filter out ultraviolet light such that only the HP Q band was irradiated, thereby preventing direct photodegradation of DPBF by UV light. After every 10 min of irradiation, the absorption spectra of the samples were monitored using a UV-Vis spectrophotometer. We then compared the photodegradation of DPBF in solutions of CoFe_2_O_4_-HP-FA particles of different sizes to evaluate the relative capacity for ^1^O_2_ production.

### 3.3. In Vitro MR-Imaging

In vitro MR-imaging of CoFe_2_O_4_-HP-FA and PC-3 cells treated with CoFe_2_O_4_-HP-FA was performed using a 3.0 Tesla (T) MR-imaging instrument (Philips Achieva X-series, Philips, Aachen, Germany). Briefly, different concentrations (0, 3.13, 6.25, 12.5, 25, and 50 μg/mL) of 60-nm CoFe_2_O_4_-HP-FA particles and PC-3 cells cultured with various concentrations (0, 3.13, 6.25, 12.5, 25, and 50 μg/mL) of 60-nm CoFe_2_O_4_-HP-FA particles for 2 h were mixed well with 1.5% agarose solution in 1.5-mL tubes. T_2_-weighted images of each sample were acquired using conventional spin echo acquisition (TR = 4000 ms, TE = 80 ms, FOV = 80 mm, slice thickness = 0.7 mm, and acquisition number = 10).

### 3.4. Biocompatibility Assessment

To evaluate the biocompatibility of different sizes of CoFe_2_O_4_-HP-FA, cytotoxicity tests were performed using L-929 and PC-3 cells as previously described [27,28,30]. Precultured cells were plated in 24-well plates (Costar Corp., Greenwich, CT, USA) at 1.0 × 10^5^ of 2.0 × 10^5^ cells/well for PC-3 and L-929 cells, respectively. The cells were then incubated at 37 °C in an atmosphere containing 5% CO_2_ for 24 h to obtain confluent monolayers of cells prior to use. After incubation, all cells were treated with various concentrations (0, 6.25, 12.5, 25, or 50 μg/mL) of CoFe_2_O_4_-HP-FA having different sizes and incubated at 37 °C in an atmosphere containing 5% CO_2_ for 24 h under dark conditions. The cells were then washed three times with PBS and incubated with Cell Counting Kit-8 solution (Dojindo Laboratories, Kumamoto, Japan) for 30 min to determine the cytotoxicity of each concentration of CoFe_2_O_4_-HP-FA. The cytotoxicity of each sample was calculated by measuring the absorbance of each well at 450 nm using a multimode microplate reader (Synergy HT; BioTek Instruments, Inc., Winooski, VT, USA). The relative cell viability is presented as the percentage of surviving cells in relation to that of untreated control cells.

### 3.5. Assessment of Photodynamic Anticancer Activity in Vitro

To evaluate the in vitro photodynamic anticancer activity of different sizes of CoFe_2_O_4_-HP-FA in cancer cells, we used PC-3 prostate cancer cells as previously described [24,25,29,30]. Briefly, the cells were incubated with different concentrations of differently sized CoFe_2_O_4_-HP-FA at 37 °C with 5% CO_2_ for 2 h to facilitate uptake of the particles into the cells under dark conditions. After incubation, the cells were irradiated using a customized green light-emitting diode (LED) equipped with a UV filter for 30 min. The LED had a wavelength range of 480–580 nm, a maximum wavelength of 515 nm, and a spot area of 20 mW. The cells were then incubated for another 24 h at 37 °C in an atmosphere containing 5% CO_2_ after irradiation with the LED. The following day, cell viability was measured using CCK-8 solution as described above to determine photodynamic anticancer activity.

### 3.6. Assessment of Cellular Uptake and Intracellular Localization of CoFe_2_O_4_-HPs-FAs

To confirm the cellular uptake and intracellular localization of CoFe_2_O_4_-HPs-FAs in prostate cancer cells, we used a transmission electron microscope (TEM) with 60 nm CoFe_2_O_4_-HPs-FAs. All samples were observed using the TEM (JEM-1011; JEOL, Tokyo, Japan) for acquisition of high-magnification images as previously described [24].

### 3.7. Apoptotic DNA Fragmentation Analysis

For DNA fragmentation analysis, apoptosis was induced by irradiation for 30 min after treatment of PC-3 cells with differently sized CoFe_2_O_4_-HP-FA at a concentration of 6.25 μg/mL. After irradiation for 30 min, cellular DNA was prepared using a Quick Apoptotic DNA Ladder Detection kit (K120-50; BioVision Inc., Milpitas, CA, USA) according to the manufacturer’s instructions. The cells were rinsed with PBS, and total DNA was extracted after the cells were lysed with Tris-EDTA lysis buffer. Total DNA was then purified with enzyme and ammonium acetate solution. Purified DNA was electrophoresed on 1.2% agarose gels at 5 V/cm for 90 min. After electrophoresis, DNA stained with Midori Green Advanced DNA stain (MG03; Nippon Genetics Europe GmbH, Duren, Germany) was analyzed using a gel documentation system (Quantum ST4; Vilber Lourmat, Eberhardzell, Germany).

### 3.8. Caspase-3 and -7 Enzyme Activity Assay

To confirm the cell death mechanism in prostate cancer cells, the activity of caspase-3 and -7 was measured using a Caspase-Glo 3/7 assay kit (G8091; Promega, Madison, WI, USA) according to the manufacturer’s instructions before and after light irradiation. Briefly, precultured PC-3 cells were incubated with different concentrations of 60-nm CoFe_2_O_4_-HP-FA at 37 °C in 5% CO_2_ for 2 h under dark conditions and were then irradiated for 30 min. Before and after 0 or 1 h of irradiation, cells were removed from the incubator and allowed to equilibrate to RT for 10 min. Next, 100 μL of Caspase-Glo reagent was added to each well, and the content of the well was gently mixed using a plate shaker at 400 rpm for 30 s. The plate was then incubated at RT for 30 min. The luminescence of each sample was measured in a multimode microplate reader (Synergy™ HT-; BioTek, Winooski, VT, USA).

### 3.9. Morphological Detection of Apoptotic Cell Death

To detect apoptotic cell death, we used an EzWay Annexin V-FITC apoptosis detection kit (K29100; Komabiotech Inc., Seoul, Korea) for detection of cell membrane inversion after irradiation. Briefly, PC-3 cells were irradiated with the LED for 30 min after treatment with 60-nm CoFe_2_O_4_-HP-FA at a concentration of 3.13 μg/mL, and the cells were stained with Annexin V-FITC reagent to stain the cell membranes and PI to stain the dead cells. The morphology of the cell membrane was observed under a laser-scanning confocal microscope (LSM 700; Carl Zeiss, Oberkochen, Germany) with a 20× objective lens and fluorescence optics (excitation at 488 nm for FITC and 530 nm for PI; emission at 518 nm for FITC and 620 nm for PI).

To evaluate apoptotic cell nuclei, Texas Red C2-maleimide (30 ng/mL in PBS; Invitrogen, Carlsbad, CA, USA) was used to stain the cell membrane, and Hoechst 33342 (1 μg/mL in PBS; Sigma-Aldrich Co., Seoul, Korea) was used to stain the nuclei. Cell morphology was analyzed using a laser-scanning confocal microscope (LSM 700) with a 20× objective lens and fluorescence optics (excitation at 595 nm for Texas Red C2-maleimide and 352 nm for Hoechst 33342; emission at 615 nm for Texas Red C2-maleimide and 620 nm for PI). Images of the cell membranes and nuclei were analyzed using ZEN imaging software (ZEN 2009; Carl Zeiss MicroImaging GmbH, Oberkochen, Germany).

### 3.10. Statistical Analysis

The data shown in this study were obtained from two independent experiments (*n* = 6) and expressed as the mean ± standard deviation (SD) for quantitative data. Statistical comparisons were performed with Student’s *t*-tests, and differences with *p* < 0.05 were considered significant.

## 4. Conclusions

In this study, we successfully designed and fabricated novel, biocompatible CoFe_2_O_4_-HP-FA particles based on PS-coated magnetic submicron particles with variable sizes for application as PDT agents in the treatment of cancer. The anticancer activity of these particles was confirmed in PC-3 cells in vitro. Additionally, we demonstrated that our CoFe_2_O_4_-HP-FA particles had high water solubility, good MRI compatibility in PC-3 cells, biocompatibility (i.e., without cytotoxic effects), and marked photodynamic anticancer activity via induction of apoptotic cell death in PC-3 cells. At the critical particle size and concentration, anticancer activity was in linear proportion with particle concentration and inversely proportional to the size of the particles. The particle size and dose effects were affected by the surface area of each particle because the number of HP molecules conjugated to the particles increased as the size decreased and the surface area increased. These results indicate that CoFe_2_O_4_-HP-FA particles may be useful in the field of PDT and could have potential as therapeutic agents for MR-imaging based on PDT due to their high saturation value for magnetization and superparamagnetism.

However, this study only used a green light to activate the HP molecules, which generated singlet oxygen to kill the cancer cells; HP molecules also have a peak in the red light band. Thus, further studies, such as in vitro and in vivo anticancer studies using red light to confirm the anticancer activity and to improve better tissue penetration for MR-imaging-based PDT, will be needed to verify the potential of these particles for clinical applications.

## Supplementary Materials

Supplementary materials can be accessed at: http://www.mdpi.com/1420-3049/21/9/1187/s1.
