# Supplementary material for: Size-Dependent Photodynamic Anticancer Activity of Biocompatible Multifunctional Magnetic Submicron Particles in Prostate Cancer Cells
Source: Molecules. 2016 Sep 6;21(9):1187. doi: 10.3390/molecules21091187 (PMC6273714; doi:10.3390/molecules21091187)
Supplement: Supplementary file 1 [file molecules-21-01187-s001.pdf]

Supplementary Materials: Particle Size-Dependent Photodynamic Anticancer Activity of Biocompatible Multifunctional Magnetic Submicron Particles in Prostate Cancer Cells

Kyong-Hoon Choi, Ki Chang Nam, Leszek Malkinski, Eun Ha Choi, Jin-Seung Jung and Bong Joo Park

To better understand the bonding nature of the carboxyl groups of HP and FA and the cations of $CoFe_2O_4$ particles, the FT-IR spectra of all samples were compared (Figure S1A,B). As reported previously [1], the IR spectrum of pure HP exhibits absorption peaks at 1704, 1424, and 1285 cm^{-1}, which correspond to the stretching modes of the free carbonyl double bond ($\upsilon_{C=O}$), the carbon-oxygen single bond (υ_{C-O}), and the O-H deformation (υ_{C-OH}), respectively (Figure S1A). These characteristic absorption peaks suggest that pure HP has protonated carboxyl groups (COOH), as expected. After the conjugation reaction occurred between the carboxyl group and the cation, the IR spectrum of the HP bonded to $CoFe_2O_4$ particles exhibited peaks corresponding to the protonated carboxyl group, and new bands appeared at 1591 and 1411 cm^{-1}, which could be ascribed to the asymmetric (υ_{as}) and symmetric (υ_{s}) stretch vibrations of the carboxylate group, respectively. The appearance of these new bands is in agreement with the results of the IR bands reported by Dravid et al. [2], who suggested that the IR bands at 1556 and 1410 cm^{-1} corresponded to the υ_{as} and υ_{s} stretching modes of the carboxylate in their oleic acid system, which showed coordination bonding between the carboxylate and the cobalt ion on the surface of the cobalt particle. Based on this result, we concluded that the carboxyl terminal groups of HP formed cation-carboxylate complexes as a result of chemical coordination bonding between the cation and the carboxylate; some molecules would also be expected to exist in the protonated carboxyl state. The IR spectrum of FA bonded to $CoFe_2O_4$ particles showed similar results after the interface bonding reaction, demonstrating that the protonated carboxyl group disappeared and that new bands appeared at 1570 and 1430 cm^{-1} (Figure S1B). Such patterns of spectral changes can also be attributed to the formation of cation-carboxylate complexes due to chemical coordination, as in the previous case.

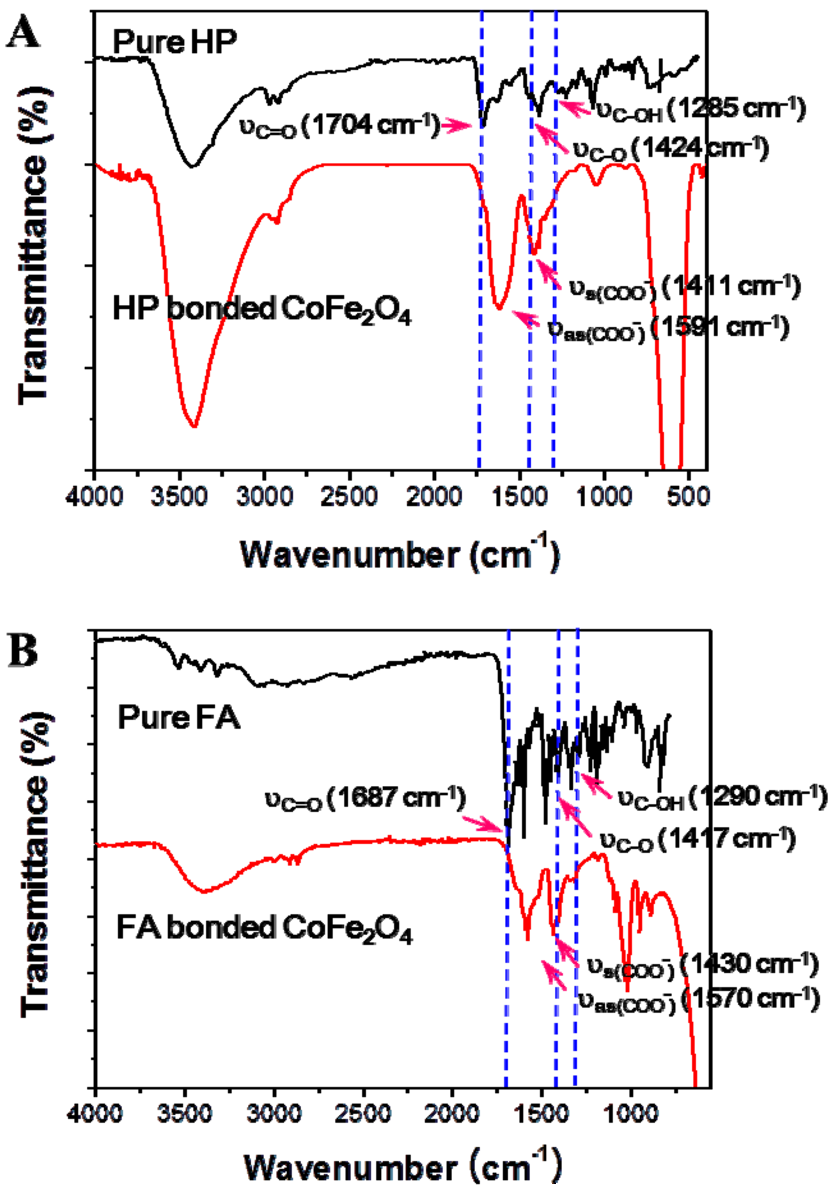

Figure S1. FT-IR spectra of (**A**) pure HP and HP bound with $CoFe_2O_4$ and of (**B**) pure FA and FA bonded with $CoFe_2O_4$.

References

1. Park, B.J.; Choi, K.-H.; Nam, K.C.; Min, J.; Lee, K.-D.; Uhm, H.S.; Choi, E.H.; Kim, H.-J.; Jung, J.-S. Photodynamic anticancer activity of $CoFe_2O_4$ nanoparticles conjugated with hematoporphyrin. *J. Nanosci. Nanotechnol.* **2015**, *15*, 7900–7906.
2. Wu, N.Q.; Fu, L., Su, M.; Aslam, M.; Wong, K.C.; Dravid, V.P. Interaction of fatty acid monolayers with cobalt nanoparticles. *Nano Lett.* **2004**, *4*, 383–386.
